# Prevalence and genotype distribution of human papillomavirus infection among women in Jingzhou, China: a population-based study of 51,720 women

**DOI:** 10.1186/s12985-023-02262-1

**Published:** 2023-12-15

**Authors:** Shun Liu, Bing Mei, Yaoling Ouyang, Chengbin Li

**Affiliations:** Department of Laboratory Medicine, Jingzhou Hospital Affiliated to Yangtze University, Jingzhou, Hubei People’s Republic of China

**Keywords:** Human papillomavirus, Prevalence, Genotype, Cervical cancer

## Abstract

**Background:**

Cervical cancer is the fourth most common cancer among women worldwide with a serious threat to women’s health. Persistent infection with high-risk human papillomavirus (HR-HPV) has been identified as the main cause of cervical cancer. This study aimed to evaluate the prevalence and genotype distribution of HR-HPV among women in Jingzhou, Hubei province, China, which is critical for the government to formulate the precision strategies of cervical cancer screening and HPV vaccine innoculation.

**Methods:**

To obtain the baseline data on the population-based prevalence and genotype distribution of HR-HPV infection among age groups and different years, a total of 51,720 women from 2018 to 2022 who went to Jingzhou Hospital Affiliated to Yangtze University for physical examination or gynacological treatment and received HR-HPV DNA genotyping were included in this retrospective study. The possible cervicovaginal infection of 15 high-risk HPV genotypes were analyzed by multiplex fluorescent real-time PCR, including HPV 16, 18, 31, 33, 35, 39, 45, 51, 52, 56, 58, 59, 66, 68 and 82.

**Results:**

The overall high-risk HPV prevalence among 51,720 women was 18.75% (9,698/51,720), and the HPV-positive rate of physical examination group (PEG) was 13.22% (541/4,091), which was lower than the HPV-positive rate of gynacological checkup group (GCG) 19.23% (9,157/47,629), with statistical difference (χ^2^ = 89.069, *P* < 0.01). The five most common prevalent genotypes were HPV52 (6.55%), HPV58 (3.41%), HPV16 (2.58%), HPV68 (1.82%) and HPV51 (1.57%). Single HPV infection was the predominant (14.36%), which compared to double (3.34%) and multiple (1.05%) infections. The HPV-positive rate was the highest in the > 60 age group (31.73%), and the lowest in the 31–40 age group (15.46%).

**Conclusions:**

The prevalence of high-risk HPV infection among women in Jingzhou area was 18.75%. HPV52, HPV58 and HPV16 genotypes were the most common. The higher prevalence was in the > 60 and ≤ 20 age group, which showed a “U” shape curve, suggesting the necessity of screening among older women to decrease the mortality of cervical cancer.

Cervical cancer is the fourth most common malignant tumor among female [1], with a serious threat to women’s health, with about 80% of new cases and 85% of deaths occurring in developing countries every year, and the incidence of cervical cancer-related deaths tends to be younger in urban China [2]. High-risk human papillomavirus (HR-HPV) persistent infection is the main cause of cervical cancer and cervical precancerous lesions [3], and HR-HPV screening is valuable for cervical cancer prevention and vaccine protection research. Previous studies have shown that there is variability in the rate of HR-HPV infection and genotypes in different regions and populations [4–6]. Therefore, understanding the characteristics of HR-HPV infection in certain region are valuable to the development of effective cervical cancer screening and HPV vaccine inoculation strategies.

As we known, most HPV infections are transient and self-limiting without a clinical symptom, while a minority persistent infection can result in precancerous cervical lesions and progress to cervical cancer eventually [7]. Therefore, early screening for HR-HPV infection is important for the prevention of cervical cancer. In this study, we collected the basic information and characteristics of HR-HPV genotypes in participants from Jingzhou Hospital Affiliated to Yangtze University and analyzed the HPV infection landscapes in different age groups, which can be important to intervene early in cervical precancerous lesions and cervical cancer, thereby reducing the incidence rate of cervical cancer in Jingzhou city.

Recently, there have been three licensed HPV vaccines, including bivalent vaccine (HPV16 and 18), quadrivalent vaccine (HPV 6, 11, 16 and 18), 9-valent vaccine (HPV 6, 11, 16, 18, 31, 33, 45, 52, and 58) [8] are approved for commercial use by National Medical Products Administration (NMPA), which inherits the supervision function from its predecessor China Food and Drug Administration (the “CFDA”), as the primary regulator for medical products, and mainly responsible for drugs (including vaccines), medical devices and cosmetics approval management. However, all these commercial vaccines cannot prevent all prevalent HPV genotypes [9]. All of these commercialized vaccines provide protection against only a few genotypes and are based on epidemiological data mainly from Western countries [10]. In order to effectively guide the use of HPV vaccine and to eliminate cervical cancer in China as much as possible, it is necessary to investigate the prevalence of HPV and its genotypes distribution in specific regions.

Jingzhou city is located in the central of China with resident population of over 5 million. So far, there is no large-scale sample study on the prevalence of human papillomavirus in Jingzhou [11], and the volume of statistics is relatively small [12]. In this study, we retrospectively analyzed the prevalence of HR-HPV and genotype distribution among women who underwent physical examination or gynacological checkup in Jingzhou Hospital Affiliated to Yangtze University from Jan 2018 to Dec 2022. We hope that results can provide a guiding basis for the development of cervical cancer screening and HPV prevention by vaccination strategies in Jingzhou area.

## Materials and methods

### Study participants

Inclusion criteria were as follows: (1) resident females in Jingzhou area, (2) non-pregnant state, (3) have a sexual history at any age, (4) no history of intravaginal medication within three days, (5) complete information on medical records. A total of 51,720 women aged between 14 and 90 years were enrolled in this study from January 2018 to December 2022. All participants were from Jingzhou Hospital Affiliated to Yangtze University. Two classification methods were used in this study: (1)The participants were divided into 6 groups according to age: ≤20 years old group (*n* = 353), 21–30 years old group (*n* = 7,036), 31–40 years old group (*n* = 14,670), 41–50 years old group (*n* = 18,379), 51–60 years old group (*n* = 9,041), > 60 years old group (*n* = 2,241). (2) All the patients were divided into two groups: the physical examination group (PEG) with 4,091 women, including women came to the Health Management Center and received routine physical examination, and gynacological checkup group (GCG) with 47,629 women, including women attended hosipital because of undiagnosed abdominal pain, genital warts, cervical intraepithelial neoplasia, irregular vaginal bleeding vaginitis, and gynaecological tumors, etc. This study was explained to each participant, and the informed consent was obtained from the participant or guardian in case of the participants under 18 years. This study was approved by Ethics Committee of Jingzhou Hospital Affiliated to Yangtze University.

### Specimen collection

Cervical exfoliated cells were collected from each participant by experienced gynecologists using the commercial conical-shaped cervical brush (Yuanjiang Medical, Ningbo, Zhejiang, China). With the help of vaginal endoscope, doctor gently turned the cervical brush clockwise for 4–5 turns to complete the sampling, and placed it into a sterile sample tube, containing 3 mL of human cell preservation medium (Yuanjiang Medical, Ningbo, Zhejiang, China). All the samples were shipped to the laboratory and then stored in a refrigerator at 2–8 ℃. HPV genotype testing was completed within 48 h.

### DNA extraction and HPV genotyping

HPV DNA was extracted from cervical samples using a commercial viral nucleic acid extraction kit (Magen, Guangzhou, China). In short, cervical cells were first digested with proteinase K. and lysis buffer. Then the released DNA was extracted by magnetic bead particles, and DNA was washed and purified from these particles using an automatic nucleic acid extractor using an automatic nucleic acid extraction instrument (Thermo Fish, America). Multiplex fluorescent real-time PCR was performed using the commercialized HR-HPV genotyping detection kit (Zhijiang Biology, Shanghai China). In brief, 5 µL of the extracted DNA was added into the 15 µL PCR master mix reaction solution. The RT-PCR parameters were: 94℃ for 2 min; 95℃ for 10 s, 62℃ for 31s (fluorescence detection), for 40 cycles. Positive and Negative controls were done throughout the process. The testing process was completed strictly in accordance to the experimental steps of the product manual, which can simultaneously detect 15 HR-HPV genotypes, including HPV 16, 18, 31, 33, 35, 39, 45, 51, 52, 56, 58, 59, 66, 68 and 82. The primers pair and TaqMan probes for 15 HR-HPV genotypes and the internal control were divided into four PCR tubes, labeled with FAM, VIC, ROX, and CY5 fluorescein respectively.

### Statistical analysis

Statistical processing SPSS (version 26.0, USA) was used for statistical analysis. All the count data were expressed as cases (n) and prevalence (%), and statistics between different groups were tested by the Chi-square (χ^2^) test, as mentioned in the previous studies [13, 14]. *P* value < 0.05 was considered statistically significant.

## Results

### Overall prevalence of HR-HPV Infection in different age groups

**Fig. 1:**
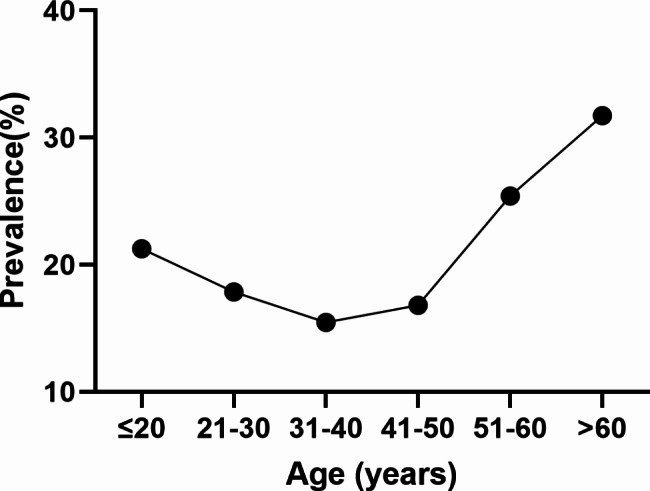
The prevalence of HR-HPV among women by age groups. Overall, the highest HR-HPV infection rates was in women aged > 60 years (31.73%), and the lowest was in the 31–40 age group (15.46%), which showed a “U” shape curve

The age-specifc prevalence of HR-HPV infection was shown in Table 1. Overall, among the 51,720 participants, the largest number of population in the 41–50 years group (35.53%, 18,379/51,720), 31–40 years group (28.36%, 14,670/51,720), and the smallest number of population in ≤ 20 years group (0.68%, 353/51,720) and > 60 years group (4.33%, 2,241/51,720) respectively. The highest infection rate was found in > 60 years group (31.73%, 711/2,241), followed by 51–60 years group (25.41%, 2,297/9,041), ≤ 20 years group ( 21.25%, 75/353), and the lowest infection rate was found in the 31–40 years group (15.46%, 2,268/14,670).There were significant differences in the prevalence of HR-HPV infection among the age groups (*χ*^*2*^=665.210, *P* < 0.01). The overall distribution among age groups showed a “U” shape, as shown in Fig. 1. The infection rates of the younger and older age groups were relatively higher, and the infection rate of the middle age group of 31–40 years was the lowest.

**Table 1:** Prevalence of HR-HPV grouped by age groups

Age group (year)	Positive cases (*n*)	Negative cases (*n*)	Total cases (*n*)	Prevalence (%)
≤ 20	75	278	353	21.25%
21–30	1257	5779	7036	17.87%
31–40*	2268	12,402	14,670	15.46%
41–50	3090	15,289	18,379	16.81%
51–60	2297	6744	9041	25.41%
> 60**	711	1530	2241	31.73%
*χ* ^*2*^	665.210			
P***	< 0.01			

*The prevalence of 31–40 years group was the lowest among the six age groups

**The prevalence of > 60 years group was the highest among the six age groups

***indicating significant differences in HR-HPV infection rates across the six age groups

### Overall prevalence of HR-HPV Infection over time

In the 51,720 cases of screening population, a total of 9,698 HR-HPV positive cases were detected, with an overall infection rates of 18.75%. The infection rates for each year from 2018 to 2022 were 20.88%, 20.25%, 20.38%, 16.88%, and 17.74%, respectively. The infection rates showed a successive decreasing trend and the comparison of the infection rates among different years was statistically difference (*χ*^*2*^ = 88.121, *P* < 0.01), as shown in Table 2.

**Table 2:** HR-HPV infection rates over time

Year	Positive cases (*n*)	Negative cases (*n*)	Total cases (*n*)	Prevalence (%)
2018	1223	4634	5857	20.88%
2019	1832	7217	9049	20.25%
2020	1810	7070	8880	20.38%
2021	2403	11,832	14,235	16.88%
2022	2430	11,269	13,699	17.74%
Total	9698	42,022	51,720	18.75%
*χ* ^*2*^	88.121			
P*	< 0.01			

*indicating significant difference of the prevalence varied across different year

### Distribution of HR-HPV genotypes in Jingzhou area

Among the 51,720 participants in Jingzhou area from 2018 to 2022, the five most common HR-HPV genotypes were HPV52 (6.55%, 3,390/51,720), HPV58 (3.41%, 1,766/51,720), HPV16 (2.58%, 1,333/51,720), HPV 68 (1.82%, 942/51,720) and HPV 51 (1.57%, 813/51,720), as shown in Table 3. The distribution of the HR-HPV genotypes varied somewhat from year to year. In 2018, 2020, 2021, and 2022, the three most common HR-HPV genotypes with the highest infection rates were HPV52, HPV58, and HPV16, over 2% of the infection rate, whereas in 2019 were HPV52, HPV68, and HPV58. Overall, the distribution of HR-HPV genotypes among screened women rankings varied considerably from year to year.

**Table 3:** Prevalence of 15 HR-HPV genotypes over time [cases (prevalence%)]

Genotypes	2018	2019	2020	2021	2022	2018–2022
HPV16	170(2.90)	281(3.11)	217(2.44)	351(2.47)	314(2.29)	1333(2.58)
HPV18	107(1.83)	121(1.34)	106(1.19)	132(0.93)	119(0.87)	585(1.13)
HPV31	51(0.87)	67(0.74)	60(0.68)	85(0.60)	65(0.47)	328(0.63)
HPV33	63(1.08)	81(0.90)	82(0.92)	106(0.74)	118(0.86)	450(0.87)
HPV35	82(1.40)	108(1.19)	58(0.65)	86(0.60)	87(0.64)	421(0.81)
HPV39	149(2.54)	163(1.80)	154(1.73)	133(0.93)	136(0.99)	735(1.42)
HPV45	33(0.56)	35(0.39)	46(0.52)	52(0.37)	43(0.31)	209(0.40)
HPV51	102(1.74)	171(1.89)	189(2.13)	174(1.22)	177(1.29)	813(1.57)
HPV52	379(6.47)	598(6.61)	626(7.05)	897(6.30)	890(6.50)	3390(6.55)
HPV56	80(1.37)	84(0.93)	123(1.39)	197(1.38)	172(1.26)	656(1.27)
HPV58	216(3.69)	323(3.57)	306(3.45)	424(2.98)	497(3.63)	1766(3.41)
HPV59	58(0.99)	98(1.08)	101(1.14)	78(0.55)	66(0.48)	401(0.78)
HPV66	93(1.59)	168(1.86)	122(1.37)	106(0.74)	122(0.89)	611(1.18)
HPV68	164(2.80)	334(3.69)	132(1.49)	154(1.08)	158(1.15)	942(1.82)
HPV82	19(0.32)	27(0.30)	16(0.18)	27(0.19)	16(0.12)	105(0.20)

### Prevalence of single and mixed HPV Infection

**Fig. 2:**
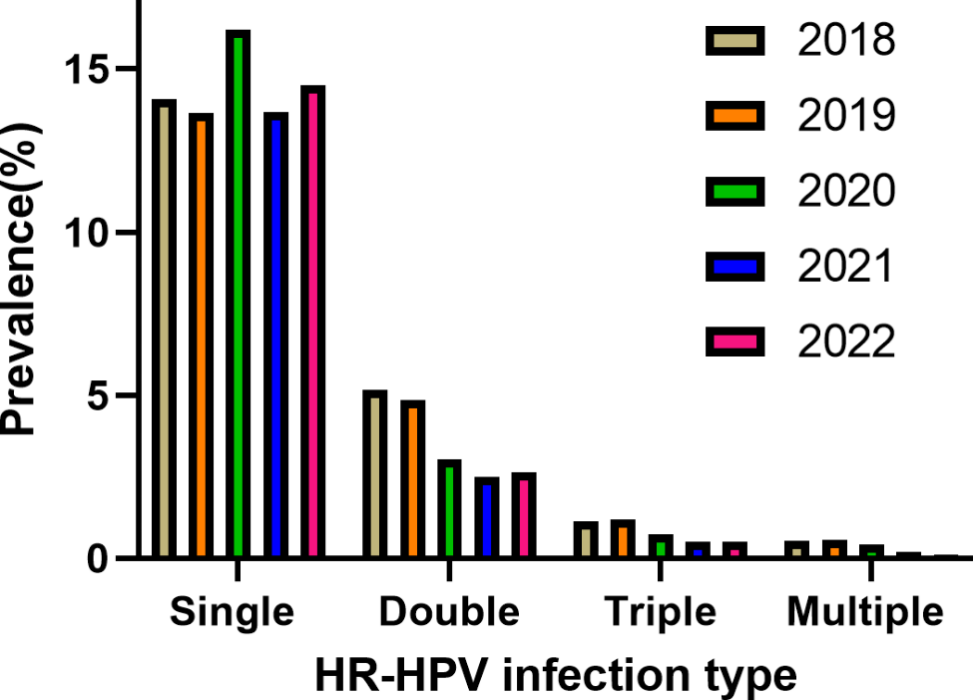
Prevalence of single and mixed HR-HPV infections over time. The prevalence of HR-HPV was predominantly single infection compared to mixed infection (*P* < 0.01)

The most frequently pattern was single infection with the prevalence of 14.36% (7,427/51,720), The prevalence of double infection (3.34%, 1,728/51,720), triple infection (0.73%, 380/51,720), and multiple infections (0.32%, 163/51,720) were relatively rare. As shown in Table 4, the prevalence of HR-HPV was predominantly single infection compared to mixed infection (*P* < 0.01). The positive rate of single infection has basically remained unchanged from 2018 to 2022, whereas the prevalence of double, triple and multiple infections have shown a decreasing trend, as shown in Fig. 2.

**Table 4:** Prevalence of single and mixed HR-HPV infections over time [cases (prevalence%)]

HPV Infection type	2018	2019	2020	2021	2022	Total
Single*	824(14.07)	1234(13.64)	1437(16.18)	1948(13.68)	1984(14.48)	7427(14.36)
Double	302(5.16)	439(4.85)	270(3.04)	355(2.49)	362(2.64)	1728(3.34)
Triple	66(1.13)	108(1.19)	66(0.74)	71(0.50)	69(0.50)	380(0.73)
Quadruple and more	31(0.53)	51(0.56)	37(0.42)	29(0.20)	15(0.11)	163(0.32)

*The prevalence of single HPV infection was higher than that of mixed HPV infections, *p* < 0.01

### Prevalence and distribution of HR-HPV Infection in PEG and GCG

All the 51,720 samples were divided into the PEG (*n* = 4,091) and the GCG (*n* = 47,629). The infection rate of GCG was 19.23% (9,157/47,629), whereas that in the PEG was 13.22% (541/4,091). As shown in Table 5, the HR-HPV infection rates of PEG was obviously lower than that GCG, which was statistically different (χ^2^ = 89.069, *P* < 0.01).

**Table 5:** The prevalence of HR-HPV infection among women in the PEG and GCG

Groups	HPV (-)	HPV (+)	Total	*χ* ^*2*^	P*
PEG	3550	541	4091	89.069	< 0.01
GCG	38,472	9157	47,629		
Total	42,022	9698	51,720		

*indicating significant difference of the prevalence between PEG and GCG group

## Discussion

Cervical cancer is a gynecological tumor that seriously endangers women’s health, with a higher incidence in developing countries and tends to be younger [2]. Globally, there are 530,000 new cases of cervical cancer and more than 270,000 deaths each year [1]. Persistent infection with HR-HPV is a major contributing factor to cervical carcinogenesis [15]. Cervical cancer is a malignant tumor whose morbidity and mortality can be controlled by clinical interventions, and the 5-year survival rate after early treatment of cervical lesions can reach 100%, while the survival rate after late treatment is only 20-30% [16]. Therefore, the screening of HR-HPV infection and genotype distribution in women is important for the prevention of HPV infection and cervical cancer.

The prevalence of HR-HPV infections and genotypes have significant regional characteristics. In this study, we retrospectively analyzed the HR-HPV infection rate of 51,720 women in Jingzhou, and the results showed that the overall infection rate was 18.75%, which was higher than that of Shannan in Tibet (8.16%) [13], Xinjiang (9.34%) [17], Shanghai (11.65%) [18] and lower than that in Zhejiang Province [5], Nanjing in Jiangsu Province [19], Heilongjiang Province (27.1%) [20], and consistent with that reported in southeastern China (18.34%) [21] and Nanning in Guangxi (18.96%) [22], which indicated that in different areas of different provinces, due to the differences in economic conditions, sexual behavioral habits and self-protection awareness, there is some variability in HPV infection rates. The infection rate of HR-HPV in the physical examination group (PEG) in this study was 13.22%, lower than that in Hangzhou, Zhejiang Province (22.41%) [23], and was basically consistent with the data released by the Vaccine and Immunization Branch of the Chinese Preventive Medicine Association for the years 2007–2018 from multiple centers [24], which showed that the total infection rate of HPV population in the general female population in China was 13.1-18.8%. Reported results of global human papillomavirus distribution vary between studies due to the differences in the regional, population, living environment, and lifestyle. The prevalence of HPV in Brazil (25.41%) [25], United States (26.8%) [26] and Eastern Europe (21.4%) [27], showed obvious different from that in China.

In this study, we have screened 9,698 HR-HPV positive cases, and the three most common genotypes were HPV52, HPV58, and HPV16, with infection rates 6.55%, 3.41%, and 2.58%, respectively, which is consistent with recent studies that reported data in Jilin province [28]. HPV 52 was the most predominant infection genotype, and the infection rate was similar to that of Shanghai [29], Wenzhou in Zhejiang province [14], Wuhan in Hubei province [30] and Xiamen in Fujian province [31], and was different from that of Hangzhou in Zhejiang province [32] and Beijing [33], where HPV 16 was the predominant infection genotype. This is also in line with the current report on the difference in the geographical distribution of HPV genotypes between the north and south of China, with HPV 16 being the predominant genotype in north China [34] and HPV 52 being the predominant genotype in south China [35].Whereas in Europe [36], sub-Saharan Africa [37] and United States [26], HPV16 was the predominant genotype, compared that HPV 52 was the most common genotype in Nigeria [38], which showed significant difference with that in China.

There is a certain correlation between the infection rate of HR-HPV and age, and the results of this study showed that the infection rate of women under 20 years old was 21.55%, and the infection rate gradually decreased with the increase of age, and the infection rate was the lowest in women aged 31–40 years old (15.46%), and it began to gradually increase in women older than 40 years old, and the highest in women older than 60 years old (31.73%). The infection rate is characterized by a “U” shaped distribution in relation to age. Some studies have pointed out that there is a U” curve between HPV infection and age [13, 31, 39], the infection rate is higher in the age groups of ≤ 20 and > 60 years old, which is consistent with the findings of the present study.The higher rate of HPV infection in women under 20 years old may be related to the fact that women have sexual intercourse at an early age, do not have a strong sense of sexual protection, and do not have a regular sexual partner. The highest rate of HPV infection in women over 60 years old may be related to endocrine changes and decreasing of immune function due to ageing, and on the other hand related to the lack of strong sexual protection measures for older women, which leads to easier infection of HPV. Therefore, it is necessary to strengthen the education of sexual safety measures, HPV infection and cervical cancer related knowledge popularization in the group of women over 60 years old, and increase HPV testing and cervical cancer screening.

The five most common HR-HPV genotypes in Jingzhou area are HPV52, HPV58, HPV16, HPV68 and HPV51. The current 9-valent HPV vaccine includes seven high-risk types, including HPV 16, 18, 31, 33, 45, 52 and 58, which can cover the three most common HR-HPV genotypes in Jingzhou area, but fails to cover the infection rate of HPV 68 and 51, indicating that the current 9-valent vaccine cannot play a fully effective role in protecting women with HR-HPV in Jingzhou. There is a need for more vaccines covering more HR-HPV genotypes to be listed on the market, or even the development of a combination vaccine for HPV genotypes in each region, which will provide more accurate protection in the region.

To summarize, HR-HPV infections in Jingzhou area are mainly single infections, and common genotypes include HPV52, 58, 16, 68, 51, etc. The 9-valent HPV vaccine currently on the market is able to cover the three most common HR-HPV genotypes, and women of appropriate age should receive 9-valent vaccine as much as possible in order to play a better immune protection role. The study focused on strengthening interventions for women under 20 and over 60 years of age, publicizing HPV infection and cervical cancer, and screening for HR-HPV infection. Our study is the first largescale sample study in Jingzhou area, which retrospectively analyzed the results of HR-HPV infection screening of more than 50,000 women in Jingzhou area, and was able to more realistically reflect the prevalence of HR-HPV infections and genotypes in Jingzhou area, which can help to provide effective references for the development of prevention and control strategies, such as vaccination, HPV genotypes testing, and cervical cancer screening.

## Conclusion

This study analyzed the prevalence of HR-HPV among women between 14 and 90 years old from 2018 to 2022 in Jingzhou area, central of China. The age-specifc prevalence of HR-HPV infection presents a “U” curve, suggesting the importance of HPV testing among younger women (≤ 20 years old) and the necessity of cervical cancer screening among older women (> 60 years old). This results will provide helpful information for precision screening of the cervical cancer and HPV vaccination in women in Jingzhou area.

## Data Availability

The data was collected from Jingzhou Hospital Affiliated to Yangtze University and the original data that support the article are available upon reasonable request.

## Abbreviations

HPV: Human papillomavirus
HR: High-risk
NMPA: National Medical Products Administration
PEG: Physical Examination Group
GCG: Gynacological Checkup Group
